# An alternative treatment for degenerative triangular fibrocartilage complex injuries with distal radioulnar joint instability: first experience with 48 patients

**DOI:** 10.1177/17531934231197942

**Published:** 2023-09-11

**Authors:** Sanharib Al Shaer, Job van der Palen, Joris Teunissen, Alexandra Fink, Brigitte van der Heijden, Oliver Zöphel, The Hand Wrist Study Group

**Affiliations:** 1 Department of Surgery, UMC Utrecht, Utrecht, The Netherlands; 2 Hand and Wrist Center, Xpert Clinics, Handtherapy, Enschede, The Netherlands; 3 Department of Epidemiology, Medisch Spectrum Twente, Enschede & Section Cognition, Data and Education, Faculty BMS, University of Twente, Enschede, The Netherlands; 4 Department of Plastic, Reconstructive and Hand Surgery, Radboud University Medical Center, Radboud Institute for Health Sciences, Nijmegen, The Netherlands; 5 Department of Plastic, Reconstructive and Hand Surgery, Jeroen Bosch Ziekenhuis ‘s-Hertogenbosch, The Netherlands; 6 Department of Plastic, Reconstructive and Hand Surgery, Ziekenhuisgroep Twente, Hengelo, The Netherlands

**Keywords:** Distal radioulnar joint instability, degenerative triangular fibrocartilage complex, ulna shortening osteotomy, extensor carpi ulnaris, patient-reported outcome measures

## Abstract

Treatment of ulnar impaction syndrome combined with distal radioulnar joint instability due to irreparable degenerative triangular fibrocartilage complex injuries can be complex. We describe the outcomes of a novel technique for restoring distal radioulnar stability due to ulnar impaction syndrome using a distally based extensor carpi ulnaris tendon strip combined with ulnar shortening osteotomy in 48 patients. Patients were assessed using standardized outcome measurements. The patient-rated wrist/hand evaluation total score improved from 66 (SD 15) at intake to 40 (SD 25) at 3 months, and 28 (SD 23) at 12 months postoperatively (*p < *0.001). Wrist extension and flexion improved significantly at 12 months from 53° (SD 11) to 65° (SD 8) (*p < *0.001) and from 45° (SD 10) to 56° (SD 12) (*p = *0.01), respectively. Adding a distally based longitudinal extensor carpi ulnaris strip to ulnar shortening osteotomy for restoring distal radioulnar joint stability seems to be an effective treatment in patients with irreparable degenerative triangular fibrocartilage complex injuries due to ulnar impaction syndrome.

**Level of evidence:** IV

## Introduction

Distal radioulnar joint (DRUJ) instability is often an underestimated or missed diagnosis in patients with ulnar-sided wrist pain. Biomechanically, the triangular fibrocartilage complex (TFCC) plays an important role in the stabilization of the DRUJ (Palmer and Werner, 1984). Missing a TFCC injury can result in progressive DRUJ instability (Thomas and Sreekanth, 2012), leading to ulnar-sided wrist pain, loss of motion and grip strength. Depending on the site and duration of traumatic peripheral TFCC injuries, the TFCC can be repaired by an open or arthroscopic approach with good results (Anderson et al., 2008; Luchetti et al., 2014). However, degenerative irreparable TFCC tears in patients with ulnar impaction syndrome (UIS) combined with DRUJ instability can be difficult to treat (Shih et al., 2000). Ulnar shortening osteotomy (USO) is widely used for the treatment of UIS, which, simultaneously, can lead to DRUJ stabilization (Fukuoka et al., 2021). However, in extended degenerative irreparable TFCC injuries, USO may not stabilize the DRUJ sufficiently. Therefore, we presume that patients with UIS-based degenerative TFCC injury combined with DRUJ dorsal instability require an additional DRUJ stabilizing technique in addition to USO. Several studies have suggested techniques to stabilize the DRUJ with variable results (Breen and Jupiter, 1989; Hermansdorfer and Kleinman, 1991; Kootstra et al., 2018; Nakamura, 2015; Shih et al., 2000; Tsai and Stilwell, 1984; Tse et al., 2013; Yeh and Shih, 2021).

We designed a modified technique to restore DRUJ instability in patients with UIS-induced irreparable degenerative TFCC, using a distally based extensor carpi ulnaris tendon (ECU) strip. This technique is added directly to the USO. The primary aim of this study was to describe this novel technique and to evaluate the patient-reported wrist function and pain 12 months postoperatively. The secondary aims were to evaluate the active range of motion, grip strength, occurrence of complications after surgery and DRUJ stability postoperatively.

## Methods

### Study design

This is a retrospective study using routinely collected data on a consecutive cohort of patients who underwent USO combined with a distally based longitudinal ECU strip between 2012 and 2020 in our national specialized hand surgery clinics in the Netherlands. All operations were performed by the senior authors (OTZ and GvC), both experts (level 5) (Tang and Giddins, 2016). All patients who visit our clinics are invited to be part of a routine outcome measurement system after their first consultation with a surgeon. Upon agreement, they receive secure web-based questionnaires at intake and at 3 months and 12 months postoperatively. The clinical and research setting of our study group has been described before (Feitz et al., 2021; Selles et al., 2020). This study is reported according to the Strengthening the Reporting of Observational Studies in Epidemiology (STROBE) guideline (von Elm et al., 2014). All patients provided written informed consent for their data to be anonymously used. The local medical ethical review committee approved this study and data collection was compliant with the principles of the Declaration of Helsinki.

### Participants

In total, 64 patients underwent USO combined with a distally based longitudinal ECU strip reconstruction during the study period. Three reminders were sent to the patients for each round of web-based questionnaires. Of them, 16 patients did not complete the questionnaires at intake or 12 months postoperatively and were excluded. The electronic patient records of the remaining 48 patients were reviewed to collect data.

### Symptoms, physical findings and additional investigations

All patients complained of ulnar-sided wrist pain. In addition, some patients complained about decreased grip strength or range of motion (ROM), which led to disabilities in their daily activities. The ulna loading tests induced pain and the DRUJ ballottement test was positive compared to the unaffected side. Additional radiological examinations were performed to evaluate fractures and ulnar variance. To confirm the diagnosis of a TFCC injury, wrist arthroscopy was performed. All patients were initially treated conservatively (e.g. hand therapy, non-steroidal anti-inflammatory drugs, bracing or corticosteroid injections) for at least 3 months. In some cases, patients had already been treated non-operatively elsewhere. Surgery was considered when conservative treatment was insufficient.

**Figure 1.**
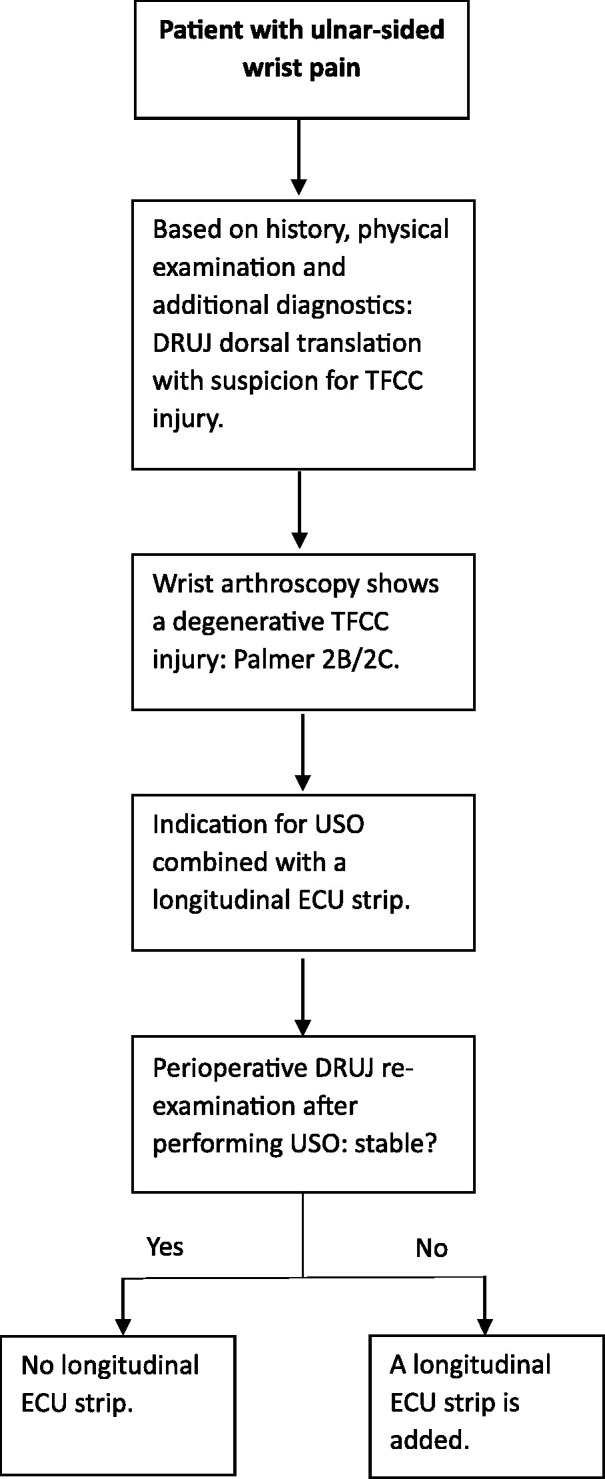
Flow chart indication for adding a longitudinal ECU strip. DRUJ: distal radioulnar joint; ECU: extensor carpi ulnaris; TFCC: triangular fibrocartilage complex; USO: ulnar shortening osteotomy.

All patients underwent a wrist arthroscopy by the same surgeon who also performed the USO combined with an ECU strip. The TFCC injuries were identified and classified according to Palmer (1989). In this study, all patients had degenerative TFCC (Palmer 2B or 2C) based on UIS and received a TFCC debridement during the arthroscopy. A USO combined with a distally based longitudinal ECU strip was performed if symptoms persisted 3 months after wrist arthroscopy. All participants did not benefit from TFCC debridement and ultimately underwent surgery. The workflow for USO combined with an ECU strip is shown in Figure 1.

### Surgical procedure

**Figure 2.**
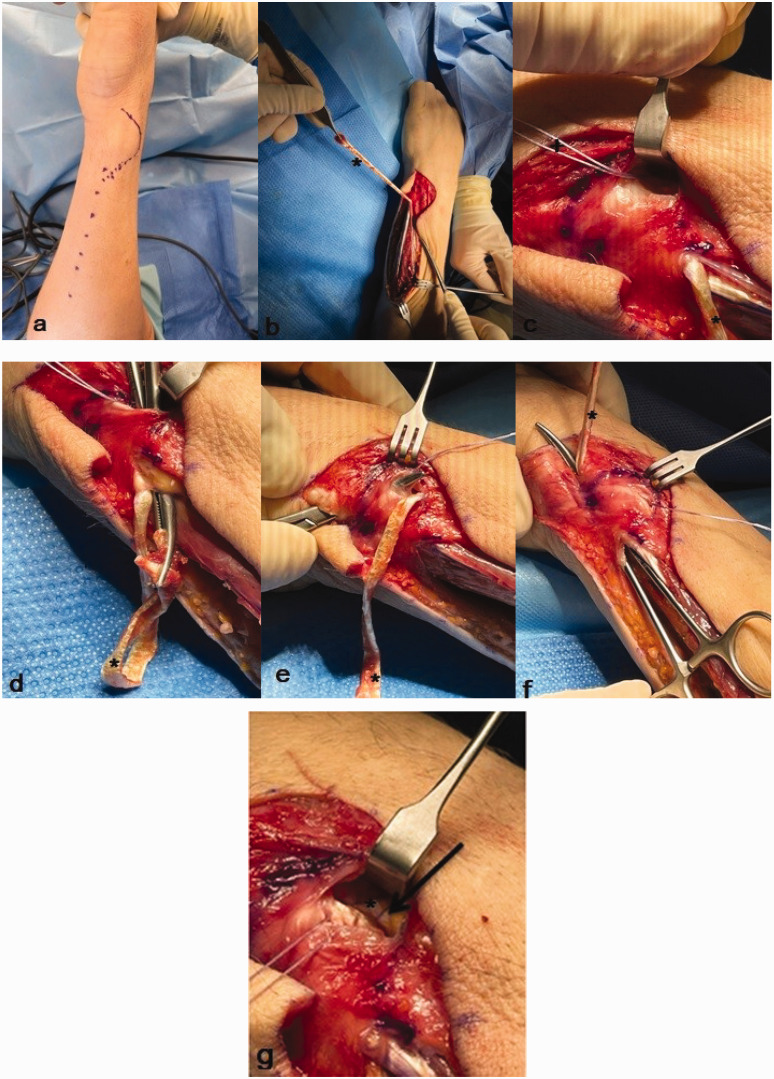
Surgical technique. (a) A dorso-ulnar incision is made. (b) An ECU strip of approximately 4 mm in diameter and 12 cm in length is used (asterisk). (c) At the level of the DRUJ, a suture anchor is placed at the dorso-ulnar surface of theContinued.radius (+). (d) The ECU strip is tunnelled over bone towards the distal radius to be to fixed with the anchor but not tight. (e) Tunnelling the strip flush over the TFCC complex towards the ulnar styloid. (f) The ECU strip is tunnelled through an anterior incision in the ECU sheath to its origin. (g) Before suturing the ends of the ECU strip. The ECU strip is sutured near the bone anchor and fixed as tight as possible to the anchor and distal radius. DRUJ: distal radioulnar joint; ECU: extensor carpi ulnaris; TFCC: triangular fibrocartilage complex; USO: ulnar shortening osteotomy.

After a brachial plexus block or general anaesthesia, a tourniquet was placed on the upper arm. A longitudinal incision on the dorso-ulnar side of the forearm was made (Figure 2a), taking care to preserve the dorsal sensory branches of the ulnar nerve. An oblique osteotomy was performed, and the ulna was shortened by several millimetres according to the preoperative planning. The preoperative planning was carried out using pronated grip views and the ulna variance was assessed with the perpendicular method (Steyers and Blair, 1989). The surgeons performed the osteotomy using an external cutting device (Acumed®, Hillsboro, OR, USA or TriMed®, Santa Clarita, CA, USA). The plate was placed on the dorsal surface of the distal ulna shaft.

**Figure 3.**
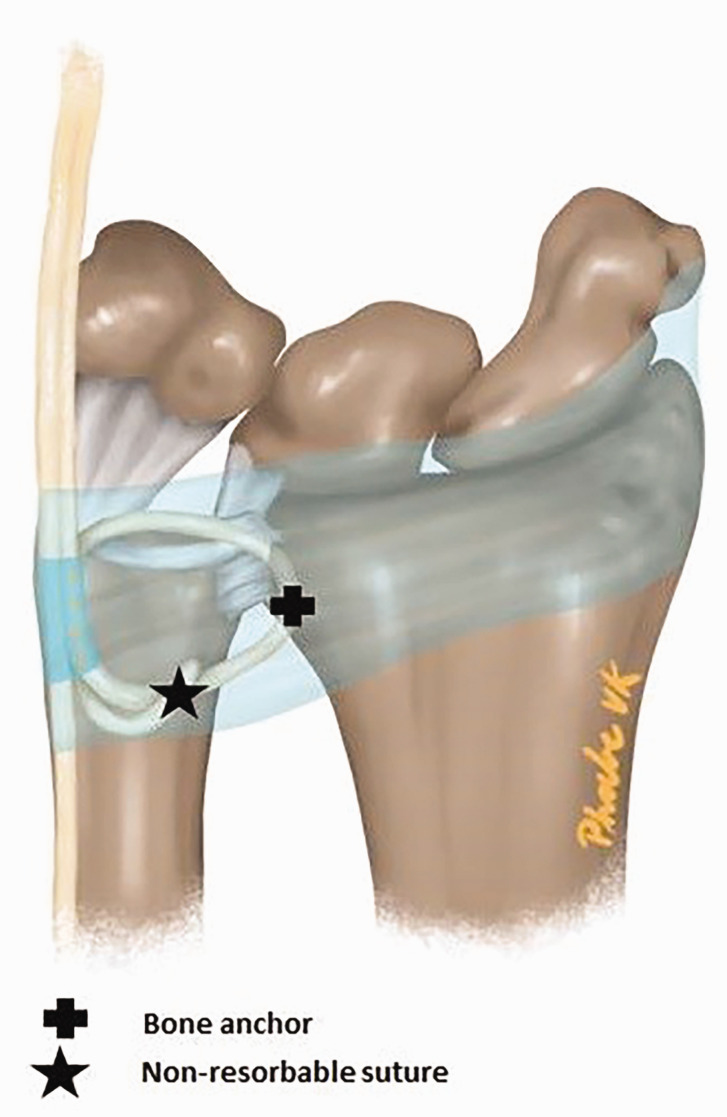
Illustration of the ECU strip technique for stabilization of the DRUJ. DRUJ: distal radioulnar joint; ECU: extensor carpi ulnaris.

After performing the USO, DRUJ stability was re-examined by the same surgeon as before the USO using a ballottement test and was compared with the unaffected side. In cases of persistent instability, an ECU strip reconstruction was added (Figure 3). Approximately 2 cm proximal from the ulnar neck, the ECU was split longitudinally up to a length of one-third of the forearm (Figure 2b). At the level of the DRUJ, a suture anchor (Parcus, Sarasota, FL, USA or DePuy Mitek anchors, Raynham, MA, USA) was placed at the dorso-ulnar area of the radius (Figure 2c). We use the radial-sided ECU strip with a diameter of 3–4 mm and a length of approximately 12–14 cm to stabilize the DRUJ. The ECU tendon strip was transposed first proximal to the ulnar head under the extensor digiti minimi flush over the bone towards the distal radius, anchoring the strip here but not too tight (Figure 2d). The next transposition of the ECU strip was made at the distal level of the ulnar head, with the strip being tunnelled flush over the TFCC complex towards the ulnar styloid (Figure 2e). At this level, an incision was made at the palmar side of the ECU sheath and the tendon strip was passed inside the ECU sheath to its origin (Figure 2f). Before suturing the ends together, we checked that the loop fitted well and was nearly tight against the ulnar head and around the DRUJ but was not yet pulled to maximal tightness. The next step was to suture the ECU strip near the bone anchor and to fix it as tight as possible to the anchor and distal radius (Figure 2g). The last step was suturing the proximal end with non-absorbable sutures. The wound was closed with Vicryl, Monocryl or Prolene (Ethicon, Guaynabo, Peurto Rico, USA) in layers.

The routine postoperative immobilization protocol consisted of a plaster cast for 3–5 days, followed by a semi-circular forearm splint (excluding the elbow) for 6 weeks. All patients were offered an extensive rehabilitation programme, including hand therapy exercises (Table 1). Postoperatively, the therapy was closely monitored and standardized by the hand therapists. Standard radiographs were taken at 3 and 12 months postoperatively to assess union of the ulnar osteotomy and additional radiographs were taken if indicated.

**Table 1.** Postoperative rehabilitation regimen after USO combined with a distally based ECU tendon strip.

Day	Postoperative regimen
0–4	Sugar-tong or Plaster-of-Paris splint
3–5	Removal of bandage, long wrist splint, tendon-gliding exercises, elbow movement, start hand therapy 2–3 times weekly
10–14	Suture removal, start scar management, oedema control, and wrist flexion and extension
42	Start pronation and supination exercises, stability training, strength training and weaning of splint

DRUJ: distal radioulnar joint; ECU: extensor carpi ulnaris; USO: ulnar shortening osteotomy.

Plate removal was considered when patients complained about hardware irritation or on patient request. Vos et al. (2012, 2013) described the indication for hardware removal in the Netherlands. The hardware was then removed if there was complete consolidation of the osteotomy on radiographs.

### Variables and data sources/measurements

Demographic variables that were routinely collected included age, sex, type of work, duration of symptoms, treatment side and hand dominance. The medical records were reviewed to collect data on treatment of the initial injury, operative variables and the occurrence of complications.

The primary outcome of this study was the Dutch-language version of Patient Rated Wrist/Hand Evaluation (PRWHE), which was administered to the patients at intake (before USO + ECU strip) and at 3 and 12 months postoperatively. According to previous studies, the PRWHE is a very responsive patient-derived questionnaire used to evaluate the treatment outcomes of UIS (MacDermid, 2019; Mehta et al., 2015; Omokawa et al., 2012). The minimal clinically important difference (MCID) in the PRWHE total score for patients who underwent USO for UIS was defined as 17 by Kim and Park (2013).

Certified hand therapists measured active range of motion (AROM) and grip strength at intake and at 3 and 12 months postoperatively. The AROM was measured in degrees using a goniometer, including extension (E), flexion (F), pronation (PRO) and supination (SUP). Grip strength was measured using an E-LINK Jamar-Style dynamometer (Biometrics, Newport, UK) according to the methods of Mathiowetz et al. (1984). The DRUJ stability was examined by the same surgeon at intake, preoperatively at the preoperative unit, intraoperatively after performing USO, after adding the ECU strip, and at 3 and 12 months postoperatively using the DRUJ ballottement test to assess translation of the ulna and compare it to the unaffected side. Pickering et al. (2022) showed that the use of the ballottement test as a primary examination technique has a positive predictive value of 81%, a negative predictive value of 55%, a specificity of 94% and sensitivity of 24% for DRUJ instability. The ballottement test was conducted with the forearm in a neutral position, in supination and pronation. All patients showed an increased dorsal translation preoperatively.

Patient satisfaction was assessed 12 months postoperatively using a digital questionnaire, by asking patients how satisfied they were with the treatment result using a Likert scale (excellent, good, adequate, moderate and poor) and whether they would undergo this procedure under the same conditions (yes or no). De Ridder et al. (2021) showed that this satisfaction assessment has a good to excellent construct validity and very high test–retest reliability in patients with hand and wrist conditions.

To score the complications, the International Consortium for Health Outcome Measurement (ICHOM) Complications in Hand and Wrist conditions (ICHAW) classification was used, which is modified from the Clavien-Dindo classification for general surgery (Clavien et al., 2009; Teunissen et al., 2022).

### Statistical analysis

Descriptive statistics were displayed as the mean and standard deviation (SD) for continuous normally distributed variables or as the median and interquartile range (IQR) for non-parametric continuous variables. Categorical variables were displayed as a number and percentage. To represent the PRWHE total score, PRWHE pain subscale, function score, AROM and grip strength over time (at intake and at 3 and 12 months postoperatively), a mixed-model analysis for repeated measures was performed. Furthermore, a non-responder analysis was performed to detect significant differences in demographics and preoperative scores between patients who completed the PRWHE total score at intake and 12 months postoperatively and patients who did not complete the PRWHE total score. A *p*-value <0.05 was considered significant.

## Results

A total of 48 patients with a mean age of 37 years (SD 13, range 17–62) were included. Demographics, details of surgery and preoperative measurements are shown in Table 2.

**Table 2.** Characteristics of the study population.

Variable	Value
No. of patients	48
Age (years)	37 (13)
Sex (female)	32 (67)
Duration of symptoms (months)	10 (4–26)
Type of work	
None	9 (19)
Light	6 (13)
Moderate	21 (44)
Heavy	12 (25)
Dominant side affected	26 (54)
Previous trauma	34 (71)
Previous surgery	10 (21)
Ulna variance	
Neutral	40 (83)
Positive	6 (13)
Negative	2 (4)
Palmer classification	
2B	26 (54)
2C	22 (46)
Type of plate for ulnar shortening	
Acumed	47 (98)
TriMed	1 (2)
Length of operation (min)	76 (19)

Data expressed as n (%), mean (SD) or median (IQR).

The non-responder analysis showed that the responders were significantly younger than the non-responders, with a mean age of 37 (SD 13) versus 45 years (SD 11) (*p* = 0.03). The responders also had significantly more pain at intake, with a mean VAS score of 35 (SD 6), compared with non-responders, who had a mean VAS score at intake of 30 (SD 6) (*p* = 0.01) according to the PRWHE subscale.

### Patient-reported pain and function

**Figure 4.**
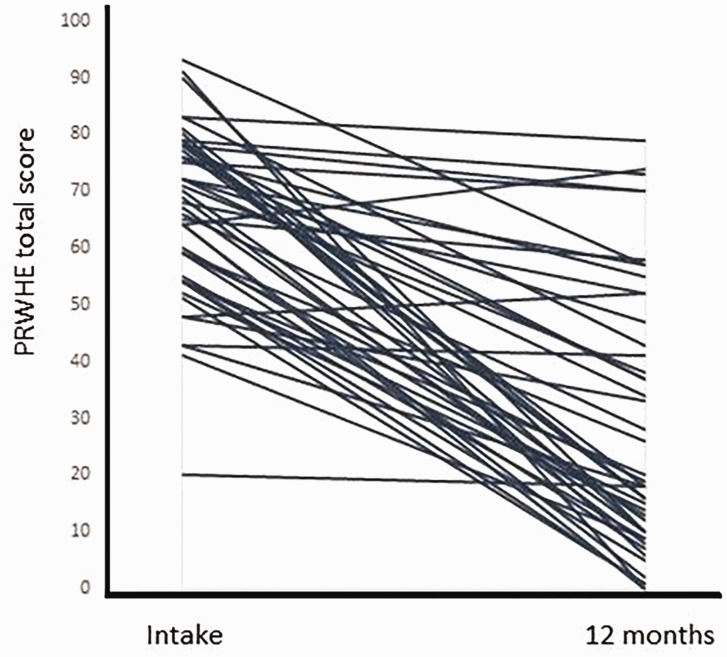
The patient-rated wrist/hand evaluation total score at intake and 12 months postoperatively plotted for each patient.

The PRWHE total score improved significantly after surgery from a mean score of 66 (SD 15) at intake to 40 (SD 25) at 3 months and 28 (SD 23) at 12 months (*p* < 0.001) (Table 3). The PRWHE pain subscale decreased significantly from 35 (SD 6) to 20 (SD 12) at 3 months and 16 (SD 12) at 12 months (*p* < 0.001) (Table 3). The mean function score improved from 33 (SD 8) to 20 (SD 13) at 3 months and 12 (SD 12) at 12 months (*p* < 0.001) (Table 3). Of the 48 patients, 12 (25%) did not improve clinically, based on the MCID of 17 points for PRWHE total score (Figure 4).

**Table 3.** Assessments at intake and at 3 and 12 months postoperatively.

Measurement	Intake (*n* = 48)	3 months (*n* = 38)	12 months (n = 48)	*p*-value*
PRWHE total score (0–100)	66 (15)	40 (25)	28 (23)	<0.001
PRWHE Pain subscale (0–50)	35 (6)	20 (12)	16 (12)	<0.001
Function (0–50)	33 (8)	20 (13)	12 (12)	<0.001
ROM (degrees)	*n* = 48	*n* = 26	*n* = 21	
Extension	53 (11)	56 (13)	65 (8)	<0.001
Flexion	45 (10)	47 (15)	56 (12)	0.010
Pronation	72 (19)	67 (13)	75 (10)	0.900
Supination	71 (17)	57 (16)	73 (10)	0.900
Grip strength (kg)	22 (12)	20 (10)	29 (11)	<0.001

Data expressed as mean (SD).

* *p*-values indicate significant change over time.

ROM: range of motion.

### Active range of motion and grip strength

Wrist extension improved significantly from 53° (SD 11) at intake to 65° (SD 8) at 12 months postoperatively (*p* < 0.001) (Table 3). Flexion also improved significantly from 45° (SD 10) to 56° (SD 12) (*p = *0.01). Pronation decreased from 72° (SD 19) at intake to 67° (SD 13) at 3 months postoperatively but recovered to its previous level of 75° (SD 10) by 12 months postoperatively. Supination showed the same trend, reducing from 71° (SD 17) at intake to 57° (SD 16) at 3 months postoperatively and then increasing again to 73° (SD 10) at 12 months postoperatively. The overall mean grip strength improved significantly from 22 kg (SD 12) to 29 kg (SD 11) at 12 months postoperatively (*p* < 0.001).

### DRUJ stability

All patients showed a reduction in DRUJ instability with stability more equivalent to the unaffected side at 3 and 12 months postoperatively.

### Patient satisfaction

Twelve months postoperatively, 58% of the patients indicated that they would undergo this procedure under the same conditions, with 19% scoring excellent, 27% good, 17% adequate, 8% moderate and 2% poor; however, 13 patients did not complete the questionnaires.

### Complications

Of all patients, 88% experienced at least one complication (Table 4). Of the 56 complications, 36 (75%) were directly related to hardware irritation and their plates were removed. There were no refractures after removal of the plates.

**Table 4.** Complications during follow-up after USO combined with longitudinal ECU split. Following ICHOM complications in Hand and Wrist conditions (ICHAW).

Complications	n (%)
None	6 (13)
Grade I	
None	31 (65)
Scar tenderness	
Expectant management	1
Analgesics	3
Hand therapy and splint	1
Ulnar nerve sensibility disturbances including numbness	
Expectant management	1
Hand therapy and splint	2
Pain only during pronation	
Analgesics	1
ECU tendinitis	
Analgesics	4
Hand therapy	1
Hand therapy and splint	1
ECU subluxation: Hand therapy and splint	1
Oedema	
Tubigrip support and hand therapy	2
Grade II	
None	46 (96)
ECU tendinitis	
Corticosteroid injection	1
Dry needling	1
Grade III	
None	12 (25)
A	0
B	
Plate irritation: Removal	36 (78)
C	0

ECU: extensor carpi ulnaris.

## Discussion

In this study, we described a modified technique, which can stabilize the DRUJ by combining USO with a distally based longitudinal ECU strip in patients with ulnar impaction syndrome and irreparable degenerative TFCC combined with DRUJ dorsal instability. The patients treated with this technique reported improved pain and wrist function 12 months postoperatively. Furthermore, the wrist extension and flexion showed a significant improvement after surgery.

Darrow et al. (1985) reported a success rate of 77% after USO in patients with ulnar-sided wrist pain caused by chronic TFCC injuries, DRUJ instability and Madelung’s deformity. Although USO itself increases DRUJ stability by ligamentotaxis, in patients with irreparable degenerative TFCC, an USO alone might not stabilize the DRUJ sufficiently. An additional stabilizing technique may be necessary. Our technique could be a good addition and is less invasive than previously described DRUJ stabilization techniques in patients with degenerative TFCC injury (Breen and Jupiter, 1989; Hermansdorfer and Kleinman, 1991; Shih et al., 2000; Tsai and Stilwell, 1984; Tse et al., 2013; Yeh and Shih, 2021). Some of these techniques involve drilling a tunnel through the radius and the ulna to insert a tendon graft (Kootstra et al., 2018; Nakamura, 2015; Shih et al., 2000; Tse et al., 2013; Yeh and Shih, 2021). By using a ECU strip, a loop can be performed to stabilize the DRUJ. The strip is partly fixed on the dorsal surface of the radius. Previously described techniques for TFCC reconstruction and stabilization appeared to tighten the DRUJ only and caused a loss of range of supination and pronation (Boyes, 1970; Sanders and Hawkins, 1989; Spinner and Kaplan, 1970). Our results showed a decrease in pronation and supination, especially the latter, during the first 3 months. This decrease may be caused by adhesions due to immobilization; however, after adequate hand therapy, recovery of the preoperative range of pronation and supination was seen.

The improvement seen in wrist function postoperatively in our study is in correspondence with those of Shih et al. (2000), who also performed a USO combined with partial ECU tendon to stabilize the DRUJ in patients with degenerative TFCC injury. The ECU tendon strip, however, was passed through a tunnel from the ulnar border to the base region of the styloid process, and then through a tunnel from the dorsal side to the palmar side of the radius near the DRUJ. In contrast to Shih et al. (2000), who assessed wrist function with the Mayo Wrist Score (MMWS), we used the PRWHE score, which has more evidence of reliability, validity and responsiveness in the evaluation of wrist function compared to other patient-reported outcome measures (Dacombe et al., 2016).

In the present study, the complication rate was 88%, of which 75% was directly related to hardware and not the ECU strip. The removal of hardware due to irritation is the most common reoperation after USO and occurs mainly 7–34 months postoperatively. The rate of hardware removal after USO is in the range of 0%–70% between studies (Fricker et al., 1996; Kitzinger et al., 2007). Controversial results are reported concerning the effect of plate location on the rate of hardware removal. Das De et al. (2015) found a significantly lower rate of plate removal in the dorsal group (1/16, 6%) compared with the palmar group (6/18, 50%), while Megerle et al. (2015) and Verhiel et al. (2020) found no significant difference based on plate location. We recently found less hardware removal when the plate was on the palmar side of the ulna (Teunissen et al., 2022), possibly due to the bulky soft tissue coverage on the palmar side. In the present study, the plate was located dorsally, which might account for the high rate of hardware removal. In addition, the ECU translates over the ulna and the plate during pronation and supination if dorsally located, thus making it more prone to irritation. Taking these findings into account, we will consider placing the plate on the palmar aspect of the ulna in the future, while reconstructing the TFCC with an ECU strip dorsally.

Eight patients had complaints initially thought to be ECU tendinitis due to narrowing of the ECU sheath as a result from tunnelling the ECU strip through the sheath. However, during the surgery for removal of the hardware in these patients, ECU tendinitis was seen at the level of the plate. After plate removal, the symptoms resolved.

Shih et al. (2000) mention three patients with complications: one with superficial wound infection and two with numbness caused by damage to the dorsal branch of the ulnar nerve. The difference in complication rate compared to Shih et al. (2000) might be explained by the fact that we used a different, stricter scoring system: the International Consortium for Health Outcome Measurement Complications in Hand and Wrist conditions (ICHAW). This is a new system, with well-described definitions of complications, designed to improve the standardization and transparency of complications registration after hand and wrist surgery.

The present study has some limitations. First, there were missing data in clinically reported measurements, such as ROM and grip strength. During the follow-up, the patient response rate decreased with postoperative time. In total, 17 patients did not attend for the 1-year measurement. Other reasons for not attending included the following: after external hand therapy (*n* = 3); reduction of medical care during the COVID-19 pandemic (*n* = 2); achievement of good wrist function and pain reduction within 1 year (*n* = 2); and hardware removal within 1 year (*n* = 3). However, there were no records of loss to follow-up due to dissatisfaction. Unfortunately, we cannot rule out dissatisfaction. Another limitation is the lack of objective measurement for DRUJ stability. Nakamura (2015) used a self-designed DRUJ instability evaluation system. Such a system could be useful to assess DRUJ stability in our follow-up studies. Finally, finding a control group was difficult. The patients in this study are so specific (patients with DRUJ instability with degenerative TFCC, Palmer 2B or 2C based on UIS) that we could not find a control group for comparison. This could be solved in the future by setting up a randomized controlled trial. Further research is also necessary to investigate the long-term outcome after USO combined with a distally based ECU strip.

This study shows that a distally based longitudinal ECU strip reconstruction carried out with USO improved wrist function and pain in patients with UIS combined with DRUJ instability due to irreparable degenerative TFCC injury. However, it remains uncertain whether the results are better than those which would be obtained with USO alone.
